# Identifying Key Drivers of Return Reversal with Dynamical Bayesian Factor Graph

**DOI:** 10.1371/journal.pone.0167050

**Published:** 2016-11-28

**Authors:** Shuai Zhao, Yunhai Tong, Zitian Wang, Shaohua Tan

**Affiliations:** Key Laboratory of Machine Perception (Ministry of Education), School of Electronics Engineering and Computer Science, Peking University, Beijing, China; East China University of Science and Technology, CHINA

## Abstract

In the stock market, return reversal occurs when investors sell overbought stocks and buy oversold stocks, reversing the stocks’ price trends. In this paper, we develop a new method to identify key drivers of return reversal by incorporating a comprehensive set of factors derived from different economic theories into one unified dynamical Bayesian factor graph. We then use the model to depict factor relationships and their dynamics, from which we make some interesting discoveries about the mechanism behind return reversals. Through extensive experiments on the US stock market, we conclude that among the various factors, the liquidity factors consistently emerge as key drivers of return reversal, which is in support of the theory of liquidity effect. Specifically, we find that stocks with high turnover rates or high Amihud illiquidity measures have a greater probability of experiencing return reversals. Apart from the consistent drivers, we find other drivers of return reversal that generally change from year to year, and they serve as important characteristics for evaluating the trends of stock returns. Besides, we also identify some seldom discussed yet enlightening inter-factor relationships, one of which shows that stocks in **Finance and Insurance** industry are more likely to have high Amihud illiquidity measures in comparison with those in other industries. These conclusions are robust for return reversals under different thresholds.

## Introduction

In the stock market, return reversal occurs when investors sell overbought stocks and buy oversold stocks, reversing the stocks’ price trends. Apart from the extensive research on market price analysis [1–7], return reversal has also attracted lots of attention. Bondt and Thaler [8, 9] initially documented long-term return reversal in the US stock market, indicating that stocks performed well in the past three to five years tend to have low future returns. Lehmann [10] and Jegadeesh [11] first recorded that short-term return reversal, specifically, weekly and monthly reversals, also exist among US stocks. Not only in US, researchers have found the phenomenon worldwide [12–16]. For instance, Chang et al. [16] provided empirical evidence on return reversal in the Japanese stock market.

A critical question about return reversal is: what are the driving forces? There are many theories proposed, however, no unanimous conclusions have been reached. Among the theories, the following ones received more supports compared with the others: 1) **overreaction hypothesis**. Overreaction hypothesis [8, 17–22] states that investors tend to overreact to recent economic developments, leading to extreme movements in stock prices in the short-run and price movements in the opposite direction in the long-run. 2) **liquidity effect**. Liquidity effect [23–27] states that the non-informational trading demanding for immediacy would drive the market prices to deviate from the fundamentals. When the non-informational trading is absorbed by liquidity suppliers, return reversal happens. 3) **January effect**. According to Tax-loss selling hypothesis [28–30], January effect [27, 29, 31, 32] states that investors tend to intensively sell stocks in December, especially those performing badly, to offset taxable realized capital gains, which induces a decline in stock prices. In January, the selling pressure disappears and investors tend to repurchase stocks, driving stock prices up. Other theories including inventory imbalances [27], lead-lag effect between large and small firms [33], and microstructure features of the stock market such as bid-ask spread [15, 34–36] have also been proposed as drivers of return reversal.

Although existing studies have generated some enlightening conclusions, they face some typical problems. First, they usually analyzed a limited number of driving factors corresponding to just one or two economic theories [24, 25, 37], leaving other driving factors deliberately neglected. Second, they generally assumed that analyzed factors are linearly correlated, which is a strong abstraction of the non-linear and time-varying characteristics of real-world market [38–43]. Third, to the best of our knowledge, they have seldom mentioned the relationships among the driving factors, which are likewise important for a more complete understanding of the mechanism in deep.

Motivated by the analysis, in this paper, we develop a new method to identify key drivers of return reversal, which is based on dynamical Bayesian factor graph. The basic structure of dynamical Bayesian factor graph is a Bayesian factor graph [44], which is a subclass of Bayesian network [45]. As a systematic non-linear and data-driven causal discovery method, Bayesian factor graph can deal with multiple factors in a unified framework, and is quite effective in uncovering factor relationships.

**Fig 1:**
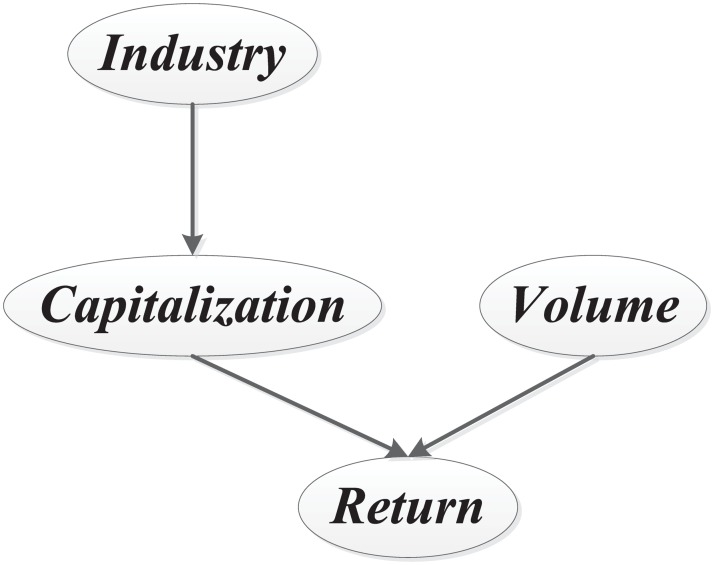
An example of Bayesian factor graph. The edge from *Industry* to *Capitalization* makes the former a parent of the latter, and the latter a child of the former. The rule is also applied to all the other edges.

Fig 1 serves as an example of Bayesian factor graph, which is associated with a small factor set *F* = {*Industry*, *Capitalization*, *Volume*, *Return*} and depicts the influential factors of the return of a stock. *Industry* stands for the industry category the stock is in. *Capitalization*, *Volume* and *Return* represent the market capitalization, trading volume and return of the stock respectively.

In Fig 1, the nodes correspond to the factors, while the edges indicate influential relationships among the factors. According to Fig 1, *Return* is causally dependent on *Capitalization* and *Volume*. *Industry* is relevant to *Capitalization*. In addition, *Industry* and *Return* are conditionally independent given *Capitalization*.

To adapt Bayesian factor graph to complex time-varying systems, we compute a time series of emergent factor graphs over a specific period of time, and term them as dynamical Bayesian factor graph [39], with which the evolution of factor relationships can be captured.

As related studies [22, 24], we merely focus on stocks with large capitalization to avoid microstructure concerns of the stock market. Additionally, we analyze the stock returns that are adjusted by the Fama-French three-factor model [46] instead of raw returns, since Blitz et al. [22] found that return reversals cleared of influences from the Fama-French factors have greater chances to make profits.

In specific, our work consists of four steps: 1) a comprehensive set of potential driving factors of return reversal corresponding to various economic theories are constructed, and the class factor indicating whether return reversal arises is defined. 2) dynamical Bayesian factor graph is applied to generate a global picture depicting factor relationships as well as their evolution. 3) from each member graph of the dynamical structure, the factors in the Markov blanket [47] of the class factor are identified as key drivers of return reversal, as conditioned on those factors the class factor is independent of any other factors. Moreover, through the mechanism of inference, the marginal probabilities and conditional probability table (CPT) of the class factor given the key drivers are calculated. By comparing the marginal and conditional probabilities, how the key drivers function in specific can be revealed. 4) some representative potential driving factors that consistently influence key reversal drivers are selected, and their influential effects on the reversal drivers are systematically studied.

The contribution of our work lies in three aspects. First, we employ dynamical Bayesian factor graph to capture the relationships as well as the dynamics of the relationships among various factors related to return reversal, from which we get a clearer picture of the mutual interaction of the financial factors. Second, based on dynamical Bayesian factor graph, we propose some definitions that are quite useful in analyzing key drivers of return reversal. These definitions aim at dealing with three problems, including evaluating the credibility of generated graphs, locating key drivers of a specific factor and capturing key driver dynamics, and figuring out how the key drivers function from a quantitative perspective. Third, through extensive experiments on the US market, we conclude that the liquidity factors consistently emerge as key drivers of return reversal, which is in line with the theory of liquidity effect. Specifically, we find that stocks with high turnover rates or high Amihud illiquidity measures [48] have a greater probability of experiencing return reversals. Apart from the consistent drivers, we find other drivers of return reversal that generally change from year to year, and they serve as important characteristics to evaluate the trends of stock returns. Besides, we also learn that among all the potential driving factors, those corresponding to overreaction hypothesis and stock industry impose most consistent influential effects on the liquidity factors. One of the influential effects shows that stocks in **Finance and Insurance** industry are more likely to have high Amihud illiquidity measures compared with those in other industries. These conclusions are robust for return reversals under different thresholds and provide insights in estimating future return reversals.

Note that there is a kind of investment strategy called contrarian strategy, the essence of which is to selectively buy stocks performing badly and sell stocks performing well with the purpose of taking advantage of return reversals to make profits [11, 13, 21, 22, 27, 49, 50]. By accurately identifying key drivers of return reversal in advance, our research can hopefully help design more profitable contrarian strategies.

The rest of the paper is organized as follows. The next section describes dynamical Bayesian factor graph and the way of identifying key drivers of return reversal in detail, followed by two sections introducing our research data and presenting empirical results based on stocks in the US market, respectively. The last section concludes the paper.

## 1 Methods

In this section, we first describe the concepts of Bayesian factor graph, then introduce dynamical Bayesian factor graph and related definitions, and finally give the way of identifying key drivers of return reversal.

### 1.1 Bayesian factor graph

Bayesian factor graph, as a subclass of Bayesian network, is a probabilistic qualitative model which is designed to uncover relationships among a set of financial factors. The edges in a factor graph reflect inter-factor relationships, including causality, relevance and conditional independence.

Described formally, a Bayesian factor graph is a directed acyclic graph in which the joint distribution of *d* factors, *X* = {*X*_1_,*X*_2_,…,*X*_*d*_}, is encoded. Nodes of the graph stand for the factors, while the graph structure reveals the qualitative information among the factors. Two unconnected nodes imply that corresponding factors are conditionally independent. If there exists an edge from node *X*_*i*_ to *X*_*j*_, then *X*_*i*_ is called a parent of *X*_*j*_, and *X*_*j*_ is a child of *X*_*i*_. The conditional probabilities of the nodes given their parents are the quantitative information of the graph.

With the parent node set of *X*_*i*_ denoted as $X_{G_{i}}$, the whole factor graph can be represented as *G* = {*G*_1_, *G*_2_, …, *G*_*d*_}, and the joint probability of *X* given *G* can be represented as:

$$p\left(X|G\right)=p\left(X_{1},\ldots ,X_{d}|G\right)=\prod_{i=1}^{d}p\left(X_{i}|X_{G_{i}}\right).\;$$ (1)

The structure of *G* is initially unknown, and needs to be learned based on the observations of *X*. In this paper, we employ incremental association Markov blanket (IAMB) algorithm [51], which is one of the optimized derivatives of inductive causation algorithm [52], to learn graph structures. The learning procedure generally comprises three steps [53]:

First, the undirected structure of a factor graph is learned by detecting the Markov blankets of factors.

During the detection process, we use the mutual information of two factors, computed by Eq (2), as the measure of factor association. Besides, we adopt Chi-square test to judge whether two factors are conditionally independent. The significance level of the independence test is set to 5%. We use the *P*-value of the test to measure the *strength* of the corresponding edge. The smaller the *P*-value is, the stronger the *strength* is.

$$MI\left(X;Y|Z\right)=\sum_{x\in X}\sum_{y\in Y}\sum_{z\in Z}p_{X,Y,Z}\left(x,y,z\right)\log\frac{p_{Z}\left(z\right)p_{X,Y,Z}\left(x,y,z\right)}{p_{X,Z}\left(x,z\right)p_{Y,Z}\left(y,z\right)}$$ (2)

In Eq (2), *p*_*X*,*Y*,*Z*_(*x*, *y*, *z*), *p*_*X*,*Z*_(*x*, *z*), *p*_*Y*,*Z*_(*y*, *z*) stand for the joint probability distribution functions of factor *X*, *Y* and *Z*, *X* and *Z*, *Y* and *Z*, respectively. *p*_*Z*_(*z*) is the marginal probability density function of *Z*. It is always true that *MI*(*X*; *Y*|*Z*) ≥ 0 and *MI*(*X*; *Y*|*Z*) = *MI*(*Y*; *X*|*Z*).

Second, set the directions of edges which are part of a *V* − *structure* in light of the *d*-separation criterion [54]. There are three kinds of basic structures in a acyclic directed graph: *X* → *Y* → *Z*, *X* ← *Y* → *Z*, and *X* → *Y* ← *Z*. The former two represent the same constraints of conditional independence that *X* and *Z* are conditionally independent given *Y*. According to Pearl and Verma [52], they are equivalent and indistinguishable based on observational data. The latter one, which is referred to as a *V*-structure, indicates that *X* is marginally independent of *Z*. Therefore, it is not equivalent to the former two structures and can be uniquely identified.

Third, add directions to other edges to meet the acyclic restriction of a factor graph.

### 1.2 Dynamical Bayesian factor graph and related definitions

Given a data set *D* = {*x*_*τ*_: *T*_0_ < *T*_1_ ≤ *τ* ≤ *T*_2_} for a factor set *X* = {*X*_1_,*X*_2_,…,*X*_*d*_} that covers the period of [*T*_0_, *T*_2_], in order to model the dynamics of the relationships among the *d* factors over [*T*_1_, *T*_2_], one Bayesian factor graph *G*_*t*_ can be built for each *t* (*T*_1_ ≤ *t* ≤ *T*_2_) based on the subset of data *D*_*t*_ = {*x*_*τ*_: *t*_0_ ≤ *τ* < *t*}. In this way, a series of discrete time *t*_*i*_, (*i* = 1, 2, …, *n*, *T*_1_ ≤ *t*_*i*_ ≤ *T*_2_) will lead to a time series of Bayesian factor graphs $G_{t_{i}}(i=1,2,\ldots ,n)$, and we term these $G_{t_{i}}$ dynamical Bayesian factor graph, which is seen as a dynamical model for *X* during [*T*_1_, *T*_2_].

Suppose we have dynamical Bayesian factor graph $G_{t}=\{G_{t_{i}}=(N,E_{i}),1\leq i\leq n\}$, where *N* is the node set of *G*_*t*_, and *E*_*i*_ is the edge set of member graph $G_{t_{i}}$. To measure how credible $G_{t_{i}}$ and *G*_*t*_ are, we propose Definition 1.

**Definition 1.** The *credibility* of $G_{t_{i}}$ and *G*_*t*_ are calculated by Eqs (3) and (4) respectively,

$$Cred\left(G_{t_{i}}\right)=\frac{\sum_{e\in E_{i}}P_{s}\left(e\right)}{|E_{i}|}$$ (3)

$$Cred\left(G_{t}\right)=\frac{\sum_{i=1}^{n}Cred(G_{t_{i}})}{n}$$ (4)

where *e* represents an edge in *E*_*i*_, |*E*_*i*_| equals the number of edges and *P*_*s*_(*e*) stands for the *P*-value of the independence test for the two factors that *e* links. As the equations show, $Cred(G_{t_{i}})$ is the average of the *P*-values for *E*_*i*_, while *Cred*(*G*_*t*_) is the average of $Cred(G_{t_{i}})(1\leq i\leq n)$. From an overall perspective, $G_{t_{i}}$ and *G*_*t*_ with smaller *credibility* values reflect more credible factor relationships.

Given the Markov blanket of a node in a factor graph, which includes its parents, its children and the children’s other parents, the node is independent of any other nodes. In other words, the Markov blanket provides all the needed information to forecast the behavior of the node. In light of the fact, we introduce Definition 2.

**Definition 2.** The nodes in the Markov blanket of node *m* (*m* ∈ *N*) in terms of $G_{t_{i}}$ are termed *key drivers* of *m* for *t*_*i*_, and denoted by $key_{t_{i}}(m)$. Given two member graphs $G_{t_{i}}$, $G_{t_{j}}\;(t_{1}\leq t_{i},t_{j}\leq t_{n},t_{i}\neq t_{j})$, the *similarity* between $key_{t_{i}}(m)$ and $key_{t_{j}}(m)$ is computed by Eq (5), which is similar to the Jaccard-index.

$$sim\left(key_{t_{i}}\left(m\right),key_{t_{j}}\left(m\right)\right)=\frac{\left|mu(key_{t_{i}}\left(m\right),key_{t_{j}}\left(m\right))\right|}{|(key_{t_{i}}\left(m\right)|+|(key_{t_{j}}\left(m\right)|-|mu\left(key_{t_{i}}\left(m\right),key_{t_{j}}\left(m\right)\right)|}$$ (5)

In Eq (5), $\left|mu(key_{t_{i}}(m),key_{t_{j}}(m))\right|$ represents the number of mutual nodes of $key_{t_{i}}(m)$ and $key_{t_{j}}(m)$.

The *similarity* measure, which varies between 0 and 1, reflects the dynamics of the *key drivers*. In specific, *similarity* = 0 indicates that two *key drivers* sets are totally different, while *similarity* = 1 means that the two sets are identical. The larger *similarity* is, the more similar the two sets are.

For a node in a factor graph, its marginal probabilities indicate the chances that the node takes possible values given no information, while its CPT conditioned on its *key drivers* indicates the chances that the node takes those values given the information provided by the *key drivers*. Suppose we concern what values of the *key drivers* would more likely lead to the node taking a specific value. We can first locate the marginal and conditional probabilities corresponding with the value, and then look up the conditional probabilities that are higher than the marginal probability. In this way, the desired *key drivers* values can be intuitively revealed. Based on the discussion, we propose the following definition.

**Definition 3.** Focusing on the situation that node *m* = *m*_*d*_ (*m* ∈ *N*), where *m*_*d*_ is a specific value that *m* can take, the marginal probability of *m* = *m*_*d*_ for *t*_*i*_, denoted by $P_{t_{i}}(m=m_{d})$, is termed *free probability*, and the conditional probabilities of *m* = *m*_*d*_ for *t*_*i*_ given $key_{t_{i}}(m)$, denoted by $P_{t_{i}}(m=m_{d}|key_{t_{i}}(m))$, are termed *driving probabilities*. Let

$$Pd_{t_{i}}\left(m=m_{d}\right)=\max_{k\in K_{t_{i}}}P_{t_{i}}\left(m=m_{d}|key_{t_{i}}\left(m\right)=k\right)$$ (6)

$$Kd_{t_{i}}\left(m=m_{d}\right)=\arg\max_{k\in K_{t_{i}}}P_{t_{i}}\left(m=m_{d}|key_{t_{i}}\left(m\right)=k\right)$$ (7)

where $K_{t_{i}}$ represents the set of values that $key_{t_{i}}(m)$ can take and *k* is one of $K_{t_{i}}$. $Pd_{t_{i}}(m=m_{d})$ and $Kd_{t_{i}}(m=m_{d})$ are termed *desired probability* and *desired values*, respectively.

The *desired probability* indicates the greatest probability that *m* = *m*_*d*_ for *t*_*i*_ given the *key drivers*, while the *desired values* show corresponding *key drivers* values. After calculating the various probabilities in Definition 3 for *t*_*i*_ (1 ≤ *i* ≤ *n*), we obtain a time series of *free probabilities* $P(m=m_{d})=\{P_{t_{i}}(m=m_{d}),1\leq i\leq n\}$, and a series of *desired probabilities* $Pd(m=m_{d})=\{Pd_{t_{i}}(m=m_{d}),1\leq i\leq n\}$. Through comparing the statistics such as the mean values of the two series, we can get an overall picture about how the *key drivers* of *m* function.

### 1.3 Dynamical Bayesian factor graph in identifying key drivers of return reversal

We first introduce the definition of return reversal, and then describe the way of identifying key drivers of return reversal using dynamical Bayesian factor graph.

#### 1.3.1 The definition of return reversal

Denoting the price, raw return, trading volume and publicly held shares of stock *i* at month *t* as *P*_*it*_, *r*_*it*_, *V*_*it*_ and $V_{it}^{{}^{\prime }}$ respectively, and calculating *r*_*it*_ through Eq (8), we define the class factor *IsReversal*_*it*_, which equals 1 or -1, to indicate whether return reversal would happen to *i* at *t* + 1 or not. For clarity, an instance where *IsReversal*_*it*_ equals 1 is termed a reversal instance hereafter.

$$r_{it}=\frac{P_{it}-P_{i,t-1}}{P_{i,t-1}}\times 100\%$$ (8)

To determine the value of *IsReversal*_*it*_, we first adjust the raw return through the Fama-French three-factor model in Eq (9):

$$r_{it}-RF_{t}=\alpha_{i}+\beta_{r}\times \left(RM_{t}-RF_{t}\right)+\beta_{s}\times SMB_{t}+\beta_{h}\times HML_{t}+rs_{it}$$ (9)

where *RM*_*t*_ and *RF*_*t*_ are market return and risk-free return at *t* respectively, and (*RM*_*t*_ − *RF*_*t*_) is the market risk factor. *SMB*_*t*_ and *HML*_*t*_ are the company size factor and value factor respectively, with *SMB* standing for “Small (market capitalization) Minus Big” and *HML* for “High (book-to-market ratio) Minus Low”. The factor values can be found at Kenneth French’s web site (http://www.mba.tuck.dartmouth.edu/pages/faculty/ken.french/data_library.html). *α*_*i*_, *β*_*r*_, *β*_*s*_ and *β*_*h*_ are parameters to be estimated, and *rs*_*it*_ is the adjusted return of *i* at *t*. As related research [22], we train the model using the data in [*t* − 36, *t* − 1] to compute *rs*_*it*_.

*IsReversal*_*it*_ will equal 1 as long as the following two restrictions are satisfied, or equal -1 otherwise.

$$rs_{it}\times rs_{i,t+1}<0$$ (10)

$$|rs_{it}-rs_{i,t+1}|>r_{th}$$ (11)

The first restriction guarantees that the return of *i* will reverse in subsequent month, while the second one rules out stochastic return reversals with threshold *r*_*th*_.

#### 1.3.2 The way of identifying key drivers of return reversal

We go through four steps to identify key drivers of return reversal.

First, we build dynamical Bayesian factor graph involving *IsReversal*_*it*_ and potential driving factors of return reversal (introduced in the next section) over a specific period of time.

Second, we evaluate the *credibility* of the dynamical structure and each of its member graphs.

Third, we identify the *key drivers* of *IsReversal*_*it*_ from the member graphs, and calculate *similarity* between the *key drivers* sets for different time to capture the dynamics of the reversal drivers.

Fourth, in terms of each member graph, we compute the *free probability*, *driving probabilities* and *desired probability* of *IsReversal*_*it*_ = 1, and identify corresponding *desired values*. After getting the time series of the *free* and *desired probabilities*, we compare the mean values of the two series to figure out how the *key drivers* affect return reversal from an overall perspective.

## 2 Data

Table 1 shows the set of potential driving factors of return reversal as well as their corresponding economic theories, types of values and short descriptions. We give some detailed explanations of the factors in S1 File.

**Table 1:** The potential driving factors of return reversal.

Economic theories	Factors	Types of values	Descriptions
Overreaction hypothesis	*HighNear*_*it*_	Continuous	The nearness of *P*_*it*_ to 5-year high
	*LowNear*_*it*_	Continuous	The nearness of *P*_*it*_ to 5-year low
Liquidity effect	*Turnover*_*it*_	Continuous	The turnover rate of *i* at *t*
	*Illiquidity*_*it*_	Continuous	The Amihud illiquidity measure of *i* at *t*
January effect	*IsDec*_*t*_	Dummy	Record whether *t* is December
Consistency effect	*PosConsis*_*it*_	Dummy	Record whether *i* experiences 4-month positive consistency at *t*
	*NegConsis*_*it*_	Dummy	Record whether *i* experiences 4-month negative consistency at *t*
Industry effect	*Industry*_*i*_	Discrete	Indicate to which industry *i* belongs
Market effect	*Efficiency*_*t*_	Continuous	A measure of market efficiency at *t*
Others	*EarnAnnDate*_*it*_	Dummy	Indicate whether the firm behind *i* releases earning announcement at *t*
	*VolGrowth*_*it*_	Continuous	The growth of trading volume of *i* at *t*

Each year from 2000 to 2010, we select 100 stocks with largest capitalization from NYSE and AMEX markets, and collect the data of *IsReversal*_*it*_ and the potential driving factors for each of the stocks. Specifically, we collect earning announcement dates of firms from the Institutional Brokers’ Estimate System, and the rest of the data from Center for Research in Security Prices. Totally, we build a panel data set containing around 13,000 instances.

Table 2 lists yearly maximum and minimum capitalization and corresponding stocks’ tickers in our data set. The unit of the capitalization is one billion dollars.

**Table 2:** Some descriptive information of our data set.

Years	Max_cap	Max_ticker	Min_cap	Min_ticker
2000	475	*GE*	29	*WM*
2001	398	*GE*	24	*COX*
2002	277	*MSFT*	19	*AA*
2003	311	*GE*	26	*BNS*
2004	386	*GE*	27	*ITW*
2005	370	*GE*	31	*FDX*
2006	447	*XOM*	35	*YHOO*
2007	512	*XOM*	39	*EMC*
2008	406	*XOM*	22	*CNQ*
2009	323	*XOM*	29	*MET*
2010	369	*XOM*	32	*DTV*

**Fig 2:**
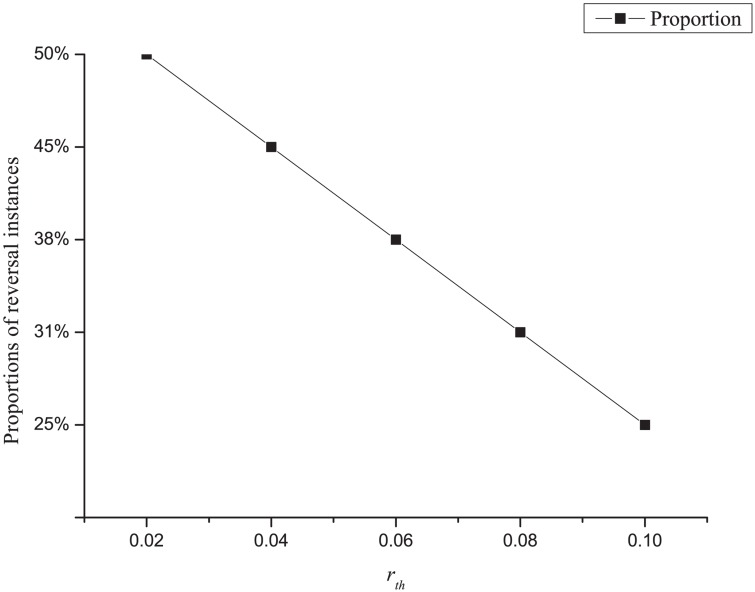
The proportions of reversal instances under different values of *r*_*th*_. As a *r*_*th*_ that is too small will lead to many random fluctuations among reversal instances, the value of the threshold should be carefully chosen. The figure can help decide what values of *r*_*th*_ should be experimented with.

To show how the quantify of reversal instances changes with *r*_*th*_, we choose five values for the threshold: 2%, 4%, 6%, 8%, and 10%, and plot corresponding proportions of reversal instances in Fig 2.

Fig 2 shows that when *r*_*th*_ ≤ 4%, over 40 percent of the instances are considered as reversal instances. When experimenting with those instances, we find no *key drivers* of *IsReversal*_*it*_ for several years, suggesting that there are many random fluctuations among the reversal instances. As a result, we focus on return reversals with *r*_*th*_ = 6%, 8% and 10% in succeeding experiments.

## 3 Results

In this section, we build dynamical Bayesian factor graph of *IsReversal*_*it*_ and the potential driving factors to identify key drivers of return reversal.

Setting *T*_0_, *T*_1_ and *T*_2_ to be the year of 2000, 2005 and 2011 respectively, we use the period [*T*_0_, *T*_1_) as the in-sample period, and [*T*_1_, *T*_2_] as the out-of-sample period. As related research [44, 55], we discretize all the continuous factors into two levels, namely high and low levels (represented by 1 and 0), through equal-frequency method.

For each year *T* in [*T*_1_, *T*_2_], a Bayesian factor graph is learned on the basis of data in [*T* − 5, *T* − 1]. During the learning process, we ban the edges pointing from *IsReversal*_*it*_ to the other factors, in that *IsReversal*_*it*_ indicates the future trends of stock returns, and thus would impossibly influence the values of the other factors at current month.

We conduct experiments using R language, and use the package ‘bnlearn’ [53] to generate network structures.

To begin with, we analyze the results of experiments for return reversal with *r*_*th*_ = 6%, and then check whether obtained conclusions are robust for return reversals under different thresholds.

### 3.1 The results of experiments with *r*_*th*_ = 6%

**Fig 3:**
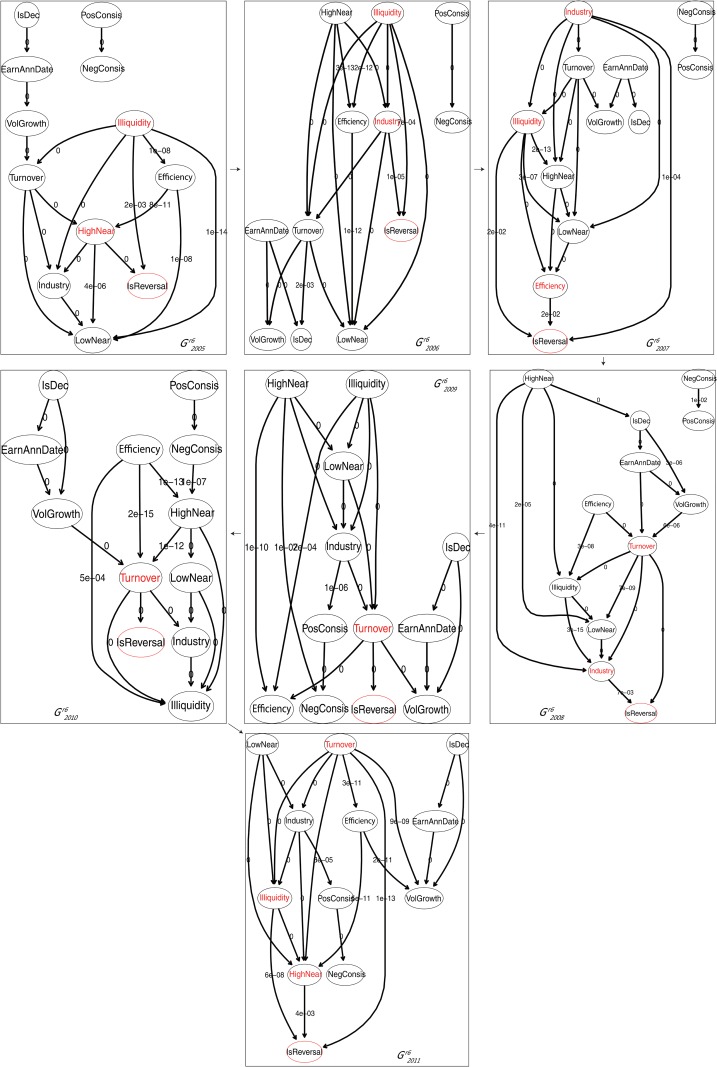
Dynamical Bayesian factor graph with *r*_*th*_ = 6%: *G*^*r*6^. The arrows indicate the time order of the member graphs. As a whole, the dynamical structure captures the mutual influential relationships as well as the dynamics of these relationships among all the factors.

Fig 3 shows the generated dynamical Bayesian factor graph $G^{r6}=\left\{G_{2005}^{r6},G_{2006}^{r6},G_{2007}^{r6},G_{2008}^{r6},G_{2009}^{r6},G_{2010}^{r6},G_{2011}^{r6}\right\}$, where the factor circled in red ellipse is the class factor and the weights on the edges are the *P*-values of corresponding independence tests. To make the graphs neater, we ignore the subscripts of all the factors. Besides, we replace the weights that are below 1e-15 (indicating edges with quite strong strength) by 0 as such weights would take too much space and intersect, making the graphs hard to read.

In Table 3, we list the *credibility* values of *G*^*r*6^ and all of its member graphs, which show that the average *P*-values of the graph edges are obviously below the significance level of 5%.

**Table 3:** The *credibility* values of *G*^*r*6^ and its member graphs.

Graphs	*Credibility*
$G_{2005}^{r6}$	1.18e-04
$G_{2006}^{r6}$	1.26e-04
$G_{2007}^{r6}$	2.14e-03
$G_{2008}^{r6}$	8.82e-04
$G_{2009}^{r6}$	5.43e-04
$G_{2010}^{r6}$	2.77e-05
$G_{2011}^{r6}$	1.89e-04
*G*^*r*6^	5.75e-04

Next, we summarize the conclusions drawn from Fig 3, in regards of identifying key drivers of return reversal, applying inference on the generated graphs and learning relationships among the potential driving factors.

#### 3.1.1 Identifying key drivers of return reversal

**Fig 4:**
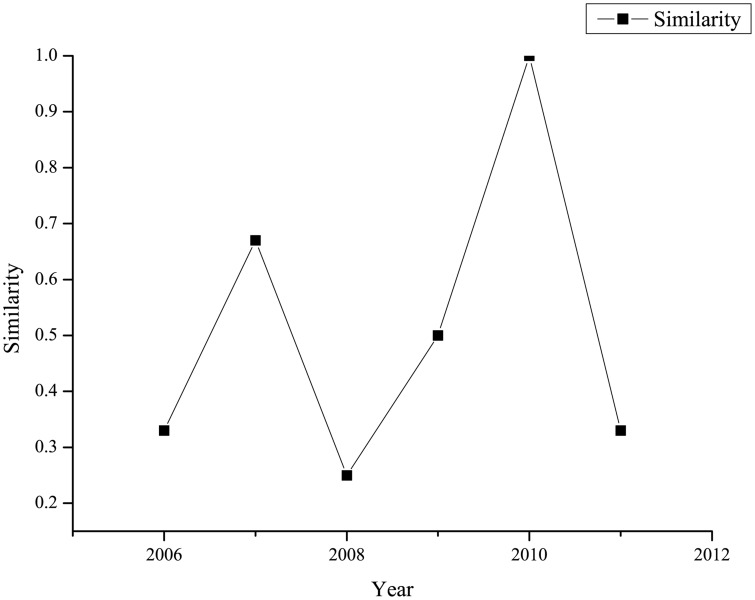
The *similarity* measures for the out-of-sample years.

First, we identify the *key drivers* of *IsReversal* from each member graph in Fig 3, and tag them in red font. In order to capture the dynamics of the *key drivers*, we calculate the *similarity* between the *key drivers* sets for two adjacent years, denoted by $S^{r6}\left(t_{i}\right)=sim\left(key_{t_{i}-1}^{r6}\left(IsReversal\right),key_{t_{i}}^{r6}\left(IsReversal\right)\right)$ (*t*_*i*_ = 2006, 2007, …, 2011). Fig 4 shows the *similarity* measures for the out-of-sample years.

From Figs 3 and 4 we learn that: 1) there are always mutual factors among the *key drivers* sets for two adjacent years, as all the *similarity* measures are above 0. As a matter of fact, the liquidity factors consistently appear as the *key drivers*, which supports the theory of liquidity effect. 2) all the *similarity* measures, except that for year 2011, are below 1, reflecting that apart from the consistent drivers, other drivers of return reversal generally change from year to year. It is worth noting that for year 2007, the factors related to three economic theories including liquidity, industry and market effects appear as the *key drivers*. 3) the factors corresponding to theories including January and consistency effects are irrelevant to return reversal during the out-of-sample period.

#### 3.1.2 Applying inference on the generated graphs

In terms of each member graph in Fig 3, the local probability distribution of each factor and the joint probability distribution of all the factors can be determined. Based on these probability distributions, we can perform various probabilistic **inferences**. The basic idea of **inference** is to monitor posterior distributions of factors of interest given some **evidence** factors [44] whose states are already known. There are some commonly used exact inference algorithms for small-scale Bayesian networks, such as variable elimination and junction tree algorithm [56].

With the inference mechanism, we calculate the *free* and *driving probabilities* of *IsReversal* = 1 (as we care more the situation that return reversal happens) by setting evidences to null and all possible states of the *key drivers* respectively. Via comparing the *free* and *driving probabilities*, how the *key drivers* function in specific can be intuitively revealed. We adopt the junction tree algorithm implemented in the R package ‘gRain’ [57] to achieve the inferences.

Regarding year 2011 as an example, Table 4 shows the *free probability* $P_{2011}^{r6}\left(IsReversal=1\right)$ and *driving probabilities* $P_{2011}^{r6}\left(IsReversal=1|key_{2011}^{r6}\left(IsReversal\right)\right)$, where $key_{2011}^{r6}\left(IsReversal\right)=\left\{Turnover,HighNear,Illiquidity\right\}$. The row in bold indicates the *desired value* $Kd_{2011}^{r6}\left(IsReversal=1\right)$ and *desired probability* $Pd_{2011}^{r6}\left(IsReversal=1\right)$.

**Table 4:** The probabilities of *IsReversal* = 1 for year 2011.

*Driving probabilities*: *key drivers* values –> probabilities	
(0,0,0)	24.8%
(0,0,1)	43.0%
(1,0,0)	40.8%
**(1,0,1)**	**45.9%**
(0,1,0)	23.5%
(0,1,1)	34.2%
(1,1,0)	37.3%
(1,1,1)	39.6%
*free probability*:	37.0%

Table 4 suggests that stocks with the following features: high turnover rates, high Amihud illiquidity measures and prices that are not near to 5-year high (*HighNear* = 0), experience return reversals with the highest probability, around 9% higher than the *free probability*.

For the other out-of-sample years, we summarize the *free* and *desired probabilities* of *IsReversal* = 1 given corresponding *desired values* in Table 5.

**Table 5:** The probabilities of *IsReversal* = 1 for the other out-of-sample years.

Years	*Desired values*	*Desired probabilities*	*Free probabilities*	*Desired*-*Free*
2005	*HighNear* = 0, *Illiquidity* = 1	43.6%	40.6%	3%
2006	*Illiquidity* = 1, *Industry* = 0	40.4%	37.2%	3.2%
2007	*Efficiency* = 1, *Illiquidity* = 1, *Industry* = 0	41.2%	34.4%	6.8%
2008	*Turnover* = 1, *Industry* = 1	39.7%	32.8%	6.9%
2009	*Turnover* = 1	39.0%	34.0%	5%
2010	*Turnover* = 1	39.8%	35.7%	4.1%
Mean		40.6%	35.8%	4.8%

Table 5 reveals the following conclusions. First, for each year, high turnover rate or high Amihud illiquidity measure signals return reversals with a greater probability. Second, for year 2007, *Efficiency* = 1 shows as part of the *desired values*, confirming that return reversal is more likely to happen when the market is comparatively inefficient. Third, on average, the *desired probabilities* are around 5% higher than the *free probabilities*, indicating that the *key drivers* we identified have great potential in predicting return reversals.

Assuming that investors have access to all the values of the *key drivers*, they can make some effective evaluations about the trends of stock returns based on the results in Tables 4 and 5. However, it is common that sometimes the *key drivers* values cannot be fully gained. In this situation, investors can still make some estimations about *IsReversal* using the inference mechanism. Let’s take the following scenario for example.

At month *t* in year 2007, investors possess evidence *e*_*i*_ regarding stock *i* shown in Table 6, and want to obtain some clues on whether *i* will experience return reversal at *t* + 1. Note that *e*_*i*_ does not cover *Illiquidity* which is in $key_{2007}^{r6}\left(IsReversal\right)$. However, it contains factors including *HighNear* and *VolGrowth* which can directly or indirectly affect *Illiquidity*.

**Table 6:** Evidence *e*_*i*_.

*HighNear*_*it*_	*Efficiency*_*t*_	*Industry*_*i*_	*EarnAnnDate*_*it*_	*IsDec*_*t*_	*VolGrowth*_*it*_
1	1	1	1	1	1

To get the clues, we adopt the junction tree algorithm to calculate the *free probability* $P_{2007}^{r6}\left(IsReversal=1\right)$ and the posterior probability $P_{2007}^{r6}\left(IsReversal=1|e_{i}\right)$. Table 7 gives the results, showing that the posterior probability is 5% higher than the *free probability*. In other words, conditioned on *e*_*i*_ stock *i* is more likely to experience return reversal compared with the case that no evidence is provided.

**Table 7:** The inference results for year 2007.

$P_{2007}^{r6}\left(IsReversal=1|e_{i}\right)$	39%
$P_{2007}^{r6}\left(IsReversal=1\right)$	34%

As Fig 3 displays, factor relationships might change over time. As a result, a same piece of evidence might lead to different inference results in different years. For instance, if we change the year in the scenario to 2011, the inference results would become those in Table 8, which imply that *e*_*i*_ leads to *i* going through return reversal with probability 5% lower than the *free probability*.

**Table 8:** The inference results for year 2011.

$P_{2011}^{r6}\left(IsReversal=1|e_{i}\right)$	32%
$P_{2011}^{r6}\left(IsReversal=1\right)$	37%

Although the inference results are not accurate enough for prediction purpose, they can provide investors with insights on what their current knowledge indicates with respect to future return reversals, and hence help them design investment strategies.

#### 3.1.3 Learning relationships among the potential driving factors

Besides key drivers of return reversal, relationships among the potential driving factors can also be conveniently studied based on Fig 3.

As we have learned that the liquidity factors consistently perform as key drivers of return reversal, subsequently we make some explorations about how the other potential driving factors influence the liquidity factors.

**Fig 5:**
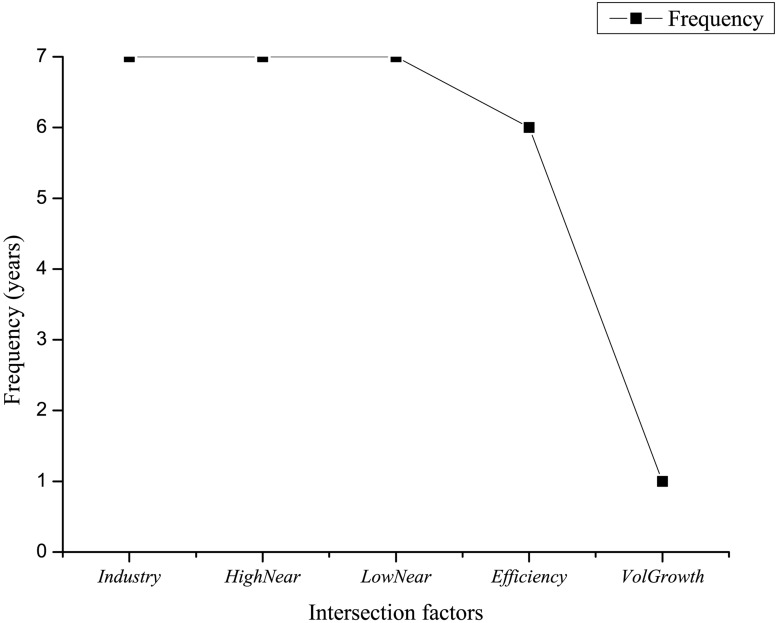
The frequencies of the intersection *key drivers* of *Illiquidity* and *Turnover*. The higher the frequency is, the more consistent the interplay between the corresponding factor and the liquidity factors is.

First, we select the representative factors that interact closely with both *Turnover* and *Illiquidity* by summarizing the intersection of the *key drivers* (except *IsReversal*) of the both factors over the out-of-sample years. Fig 5 displays the frequencies of the intersection factors.

Fig 5 shows that *HighNear*, *LowNear* as well as *Industry* which respectively represent the theories of overreaction hypothesis and industry effect impose influential effects on the liquidity factors over the whole out-of-sample period, and thus are chosen as the representative factors.

Second, we use the junction tree algorithm to calculate the *free* and conditional probabilities of *Turnover* and *Illiquidity*, conditioned respectively on (*HighNear*, *LowNear*) and *Industry*. As high turnover rate or high Amihud illiquidity measure is more likely to signal return reversal, we merely focus on the *free* and highest conditional probabilities of *Turnover* = 1 and *Illiquidity* = 1. Tables 9 and 10 show the results given (*HighNear*, *LowNear*), while Tables 11 and 12 list those given *Industry*.

**Table 9:** The *free* and highest conditional probabilities of *Turnover* = 1 given (*HighNear*, *LowNear*).

Years	(*HighNear*,*LowNear*)	Conditional probabilities	*Free probabilities*	Conditional-*Free*
2005	(0,0)	49.0%	33.9%	15.1%
2006	(0,0)	51.1%	34.5%	16.6%
2007	(0,0)	54.9%	36.2%	18.7%
2008	(0,0)	54.4%	41.1%	13.3%
2009	(0,0)	56.3%	49.9%	6.4%
2010	(0,0)	64.8%	60.2%	4.5%
2011	(0,0)	74.6%	68.4%	6.2%
Average		57.9%	46.3%	11.5%

**Table 10:** The *free* and highest conditional probabilities of *Illiquidity* = 1 given (*HighNear*, *LowNear*).

Years	(*HighNear*,*LowNear*)	Conditional probabilities	*Free Probabilities*	Conditional-*Free*
2005	(0,1)	71.6%	68.3%	3.3%
2006	(0,1)	64.2%	60.3%	3.9%
2007	(0,1)	56.7%	51.6%	5.0%
2008	(0,0)	45.6%	42.1%	3.5%
2009	(1,0)	42.6%	38.0%	4.6%
2010	(0,1)	43.3%	36.9%	6.3%
2011	(0,1)	47.1%	34.3%	12.8%
Average		53.0%	47.4%	5.6%

**Table 11:** The *free* and highest conditional probabilities of *Turnover* = 1 given *Industry*.

Years	Industry	Conditional probabilities	*Free probabilities*	Conditional-*Free*
2005	0	41.9%	33.9%	8.0%
2006	0	43.7%	34.5%	9.2%
2007	0	48.5%	36.2%	12.3%
2008	0	54.7%	41.1%	13.6%
2009	0	64.5%	49.9%	14.7%
2010	0	75.5%	60.2%	15.3%
2011	0	82.2%	68.4%	13.9%
Average		58.7%	46.3%	12.4%

**Table 12:** The *free* and highest conditional probabilities of *Illiquidity* = 1 given *Industry*.

Years	Industry	Conditional probabilities	*Free probabilities*	Conditional-*Free*
2005	2	73.8%	68.3%	5.6%
2006	2	66.1%	60.3%	5.8%
2007	2	58.3%	51.6%	6.6%
2008	2	51.1%	42.1%	9.0%
2009	2	49.8%	38.0%	11.8%
2010	2	50.8%	36.9%	13.8%
2011	2	49.8%	34.3%	15.5%
Average		57.1%	47.4%	9.7%

From Tables 9 and 10, we learn that: (1) stocks whose prices are neither near to 5-year high nor near to 5-year low tend to have high turnover rates with greater probabilities, averagely around 12% higher than the *free probabilities*. (2) for all the out-of-sample years except 2008 and 2009, stocks whose prices are near to 5-year low are more likely to have high Amihud illiquidity measures, with probabilities around 6% higher than the *free probabilities* on average.

Tables 11 and 12 suggest that: (1) stocks in other industries (not **Manufacturing** and **Finance and Insurance**) tend to have high turnover rates with greater probabilities, around 13% higher than the *free probabilities* averagely. (2) stocks in **Finance and Insurance** industry are more likely to have high Amihud illiquidity measures, with probabilities around 10% higher than the *free probabilities* on average.

To the best of our knowledge, few efforts have been made to systematically study the inter-factor relationships above. These relationships can be quite helpful for investors in estimating future return reversals when the values of the liquidity factors are unavailable.

### 3.2 Robustness check

To check the robustness of the following two main conclusions in the previous subsection, in this subsection, we experiment on return reversals with *r*_*th*_ = 8%, 10% respectively.

**Conclusion 1.** The liquidity factors consistently serve as key drivers of return reversal, and other drivers generally change from year to year.

**Conclusion 2.** Stocks with high turnover rates or high Amihud illiquidity measures experience return reversals with a greater probability.

S1 and S2 Figs give corresponding dynamical Bayesian factor graphs, while S1 and S2 Tables show the *credibility* of the dynamical structures and their member graphs. From the tables we learn that the factor relationships reflected by the graphs are quite credible. The *key drivers* of *IsReversal* are tagged in red font. S3 and S4 Figs display the *similarity* measures between the *key drivers* sets for two adjacent years.

S1, S2, S3 and S4 Figs intuitively confirm that Conclusion 1 still stands for return reversals with *r*_*th*_ = 8%, 10%. Moreover, it is worth noting that the *key drivers* under a lower *r*_*th*_ generally constitute part, or all of the *key drivers* under a higher *r*_*th*_. For example, for year 2009, the *key drivers* only include *Turnover* when *r*_*th*_ = 6%, which extend to *Turnover* and *Industry* when *r*_*th*_ = 8%, and to *Turnover*, *Industry* and *Illiquidity* when *r*_*th*_ = 10%.

S3 and S4 Tables show the *free* and *desired probabilities* of *IsReversal* = 1 given corresponding *desired values* for the out-of-sample years. It is obvious that both tables support Conclusion 2. What’s more, the tables also indicate that with *r*_*th*_ raised, the mean values of both the *free* and *desired probabilities* turn lower, which appears reasonable, whereas the differences between them go up. This phenomenon implies that for return reversals under more restrictive conditions, the influential effects imposed by the *key drivers* become more obvious.

As to relationships among the potential driving factors, many such relationships in the previous subsection, such as those between *Industry* and the liquidity factors, vary little, in that all the factor values except those of *IsReversal* used in this subsection remain unchanged. For clarity, we do not give detailed analyses.

In summary, we conclude that the main conclusions for return reversal with *r*_*th*_ = 6% are robust for return reversals with *r*_*th*_ = 8%, 10%.

## 4 Conclusions

In this paper, we employ dynamical Bayesian factor graph to identify key drivers of return reversal. Our empirical results demonstrate that liquidity factors consistently emerge as key drivers of return reversal, supporting the theory of liquidity effect. In specific, stocks with high turnover rates or high Amihud illiquidity measures experience return reversals with a greater probability. Apart from liquidity factors, other drivers of return reversal generally change from year to year. We also learn that factors corresponding to overreaction hypothesis and stock industry impose most consistent influential effects on liquidity factors. One of the influential effects shows that stocks in **Finance and Insurance** industry are more likely to have high Amihud illiquidity measures compared with those in other industries. These conclusions are robust for return reversals under different thresholds. Our work reveals the drivers of return reversal from a more comprehensive perspective and sheds light on designing more profitable contrarian investment strategies.

Although our research has generated some enlightening results, there is room for improvements. Currently, we only study stocks in the US market based on discretized factors. In the coming research, we would study stocks in international markets with continuous factors analyzed.

## Supporting Information

S1 Fig Dynamical Bayesian factor graph with *r*_*th*_ = 8%: *G*^*r*8^. (EPS)

S2 Fig Dynamical Bayesian factor graph with *r*_*th*_ = 10%: *G*^*r*10^. (EPS)

S3 Fig The *similarity* measures for the out-of-sample years with *r*_*th*_ = 8%. (EPS)

S4 Fig The *similarity* measures for the out-of-sample years with *r*_*th*_ = 10%. (EPS)

S1 Table The *credibility* of *G*^*r*8^ and its member graphs. (PDF)

S2 Table The *credibility* of *G*^*r*10^ and its member graphs. (PDF)

S3 Table The probabilities of *IsReversal* = 1 with *r*_*th*_ = 8% for the out-of-sample years. (PDF)

S4 Table The probabilities of *IsReversal* = 1 with *r*_*th*_ = 10% for the out-of-sample years. (PDF)

S1 File Some detailed explanations of the potential driving factors of return reversal. (PDF)

S1 Data The capitalization of the stocks in our data set. (XLSX)

S2 Data The panel data set that is used in our experiments. (XLSX)
