# Supplementary material for: Identifying Key Drivers of Return Reversal with Dynamical Bayesian Factor Graph
Source: PLoS One. 2016 Nov 28;11(11):e0167050. doi: 10.1371/journal.pone.0167050 (PMC5125680; doi:10.1371/journal.pone.0167050)
Supplement: S1 Table — (PDF) [file pone.0167050.s005.pdf]

The *credibility* of G^{r8} and its member graphs

Graphs	*Credibility*
G^{r8}_{2005}	2.50e-04
G^{r8}_{2006}	1.90e-04
G^{r8}_{2007}	7.04e-06
G^{r8}_{2008}	6.05e-06
G^{r8}_{2009}	8.10e-04
G^{r8}_{2010}	1.37e-03
G^{r8}_{2011}	9.14e-06
G^{r8}	3.77e-04
