# Supplementary material for: Identifying Key Drivers of Return Reversal with Dynamical Bayesian Factor Graph
Source: PLoS One. 2016 Nov 28;11(11):e0167050. doi: 10.1371/journal.pone.0167050 (PMC5125680; doi:10.1371/journal.pone.0167050)
Supplement: S2 Table — (PDF) [file pone.0167050.s006.pdf]

The *credibility* of G^{10} and its member graphs

Graphs	*Credibility*
G^{r10}_{2005}	1.05e-04
G^{r10}_{2006}	1.07e-05
G^{r10}_{2007}	1.01e-05
G^{r10}_{2008}	5.70e-06
G^{r10}_{2009}	7.54e-04
G^{r10}_{2010}	7.68e-05
G^{r10}_{2011}	6.93e-05
G^{r10}	1.47e-04
