# Supplementary material for: Identifying Key Drivers of Return Reversal with Dynamical Bayesian Factor Graph
Source: PLoS One. 2016 Nov 28;11(11):e0167050. doi: 10.1371/journal.pone.0167050 (PMC5125680; doi:10.1371/journal.pone.0167050)
Supplement: S3 Table — (PDF) [file pone.0167050.s007.pdf]

The probabilities of $IsReversal = 1$ with $r_{th} = 8\%$ for the out-of-sample years

Years	*Desired values*	*Desired probabilities*	*Free probabilities*	*Desired-Free*
2005	$HighNear = 0, Illiquidity = 1$	37.7%	34.2%	3.5%
2006	$Turnover = 1, HighNear = 0, Industry = 1$	43.2%	30.0%	13.2%
2007	$Illiquidity = 1, Industry = 0$	32.3%	26.5%	5.8%
2008	$Turnover = 1, Industry = 1$	33.0%	24.6%	8.4%
2009	$Turnover = 1, Industry = 1$	33.6%	26.2%	7.4%
2010	$Illiquidity = 1, Turnover = 1, HighNear = 0, Industry = 2$	51.2%	28.5%	22.7%
2011	$Turnover = 1, HighNear = 0, Illiquidity = 1$	41.7%	29.8%	11.9%
	Mean	39.0%	28.5%	10.5%
