# Supplementary material for: Identifying Key Drivers of Return Reversal with Dynamical Bayesian Factor Graph
Source: PLoS One. 2016 Nov 28;11(11):e0167050. doi: 10.1371/journal.pone.0167050 (PMC5125680; doi:10.1371/journal.pone.0167050)
Supplement: S4 Table — (PDF) [file pone.0167050.s008.pdf]

The probabilities of $IsReversal = 1$ with $r_{th} = 10\%$ for the out-of-sample years

Table 1

Years	*Desired values*	*Desired probabilities*	*Free probabilities*	*Desired-Free*
2005	$Illiquidity = 1, HighNear = 0, Industry = 1$	33.8%	28.1%	5.7%
2006	$Turnover = 1, HighNear = 0, Industry = 1$	37.9%	23.5%	14.4%
2007	$Illiquidity = 1, HighNear = 0, Industry = 0$	28.4%	20.2%	8.2%
2008	$Illiquidity = 1, Turnover = 1, Industry = 1$	31.0%	18.2%	12.8%
2009	$Illiquidity = 1, Turnover = 1, Industry = 1$	32.8%	20.4%	12.4%
2010	$Illiquidity = 1, Turnover = 1, HighNear = 0, Industry = 2$	45.3%	23.0%	22.3%
2011	$Illiquidity = 1, Turnover = 1, HighNear = 0, Industry = 2$	44.6%	23.7%	20.9%
Mean		36.3%	22.4%	13.9%
