# Supplementary material for: Identifying Key Drivers of Return Reversal with Dynamical Bayesian Factor Graph
Source: PLoS One. 2016 Nov 28;11(11):e0167050. doi: 10.1371/journal.pone.0167050 (PMC5125680; doi:10.1371/journal.pone.0167050)
Supplement: S1 File — (PDF) [file pone.0167050.s009.pdf]

# Some detailed explanations of the potential driving factors of return reversal

Denoting the price, raw return, trading volume and publicly held shares of stock $i$ at month $t$ as $P_{it}$, $r_{it}$, $V_{it}$ and $V_{it}^{'}$ respectively, we give detailed explanations of the potential driving factors as follows.

$\boldsymbol{HighNear_{it}}$, $\boldsymbol{LowNear_{it}}$. Stocks whose prices are more nearer to 5-year high (5-year low) are prone to experience return reversals based on **overreaction hypothesis** [1]. Accordingly, we define $HighNear_{it}$ and $LowNear_{it}$, which are calculated by Eqs (1) and (2) respectively. Given $HighNear_{it} \leq 1$ ($LowNear_{it} \leq 1$), the larger $HighNear_{it}$ ($LowNear_{it}$) is, the nearer is $P_{it}$ to 5-year high (5-year low). $HighNear_{it}$ ($LowNear_{it}$) above 1 means that $P_{it}$ is higher (lower) than 5-year high (5-year low).

$$HighNear_{it} = \frac{P_{it}}{\max\left\{P_{i,t-j}\right\}_{j=1,2,\ldots,60}} \tag{1}$$

$$LowNear_{it} = \frac{\min\left\{P_{i,t-j}\right\}_{j=1,2,\ldots,60}}{P_{it}} \tag{2}$$

$\boldsymbol{Illiquidty_{it}}$, $\boldsymbol{Turnover_{it}}$. Standing for turnover rate and Amihud illiquidity measure [2] of $i$ at $t$, and calculated by Eqs (3) and (4) respectively, $Illiquidty_{it}$ and $Turnover_{it}$ are widely adopted when **liquidity effect** of return reversal is analyzed [3–5].

$$Illiquidty_{it} = \frac{1}{n}\sum_{d=1}^{n}\frac{|r_{id}|}{P_{id} \times N_{id}} \tag{3}$$

$P_{id}$, $N_{id}$, $|r_{id}|$ stand for the close price, number of traded shares and absolute raw return of $i$ at day $d$, and $n$ equals the number of trading days in $t$.

$$Turnover_{it} = \frac{V_{it}}{V_{it}^{'}} \times 100\% \tag{4}$$

It is noteworthy that $Illiquidty_{it}$ and $Turnover_{it}$ measure different aspects of stock liquidity, hence can coexist in the analysis [4, 6].

$\boldsymbol{IsDec_{t}}$. Taking **January effect** of return reversal [7] into consideration, we use $IsDec_t$, which equals 1 or 0, to record whether $t$ is December or not.

$\boldsymbol{PosConsis_{it}}$, $\boldsymbol{NegConsis_{it}}$. Studies including [8, 9] claimed that short-run consistency is closely related to return reversal. In light of the conclusion, we define $PosConsis_{it}$ ($NegConsis_{it}$) to represent four-month positive (negative) consistency of $i$ at $t$. Similar to [10], $PosConsis_{it}$ ($NegConsis_{it}$) will be set to 1 if more than three among $rs_{it}$, $rs_{i,t-1}$, $rs_{i,t-2}$, $rs_{i,t-3}$ are positive (negative), or to 0 otherwise.

$\boldsymbol{EarnAnnDate_{it}}$. Considering studies such as [11] and [12] found that stocks go through return reversals with a higher probability when firm-specific news is released, we use $EarnAnnDate_{it}$, which equals 1 or 0, to indicate whether the firm behind $i$ releases earning announcement at $t$ or not.

$\boldsymbol{VolGrowth_{it}}$. Extant research revealed that stock trading volume is relevant to return reversal [11, 13–16], hence we let $VolGrowth_{it}$, calculated by Eq (5), to represent the growth of trading volume of $i$ at $t$.

$$VolGrowth_{it} = \frac{V_{it} - V_{i,t-1}}{V_{i,t-1}} \times 100\% \tag{5}$$

***$Industry_i$.*** As existing studies [12, 17] found that return reversal is more dramatic within, instead of across industries, we use $Industry_i$ to record the industry to which $i$ belongs based on the Standard Industrial Classification system (Specific information is available at: `https://www.osha.gov/pls/imis/sic_manual.html`). Observing that most stocks in our data set fall in **Finance and Insurance**, and **Manufacturing** industries, we will set $Industry_i$ to 1 if $i$ belongs to **Manufacturing** industry, or to 2 if $i$ belongs to **Finance and Insurance** industry, or to 0 otherwise.

***$Efficiency_t$.*** Scholars have pointed out that return reversal is more likely to happen when market is inefficient [18], so we design $Efficiency_t$, calculated by Eq (6), to evaluate the efficiency of the US market at $t$.

$$Efficiency_t = |\frac{1}{N_t}\sum_{d=1}^{N_t} HE_d - 0.5| \tag{6}$$

In Eq (6), $N_t$ is the number of trading days in $t$, and $HE_d$ represents day $d$'s value of Hurst exponent [19], which is a widely used measure for stock market efficiency [20]. Specifically, $HE_d$ varies between 0 and 1, and is computed based on daily raw returns of S&P 500 index in $[d - 199, d]$ through the R/S method. The larger $Efficiency_t$ is, the more inefficient the market is at $t$.

## References

1. George TJ, HWANG CY. Long-Term Return Reversals: Overreaction or Taxes? The Journal of finance. 2007;62(6):2865–2896.
2. Amihud Y. Illiquidity and stock returns: cross-section and time-series effects. Journal of Financial Markets. 2002;5(1):31–56.
3. Avramov D, Chordia T, Goyal A. Liquidity and autocorrelations in individual stock returns. The Journal of finance. 2006;61(5):2365–2394.
4. Hameed A, Huang J, Mian GM. Industries and stock return reversals. Journal of Financial and Quantitative Analysis. 2015;50(1-2):89–117.
5. Nagel S. Evaporating liquidity. Review of Financial Studies. 2012;25(7):2005–2039.
6. De Groot W, Huij J, Zhou W. Another look at trading costs and short-term reversal profits. Journal of Banking & Finance. 2012;36(2):371–382.
7. D'Mello R, Ferris SP, Hwang CY. The tax-loss selling hypothesis, market liquidity, and price pressure around the turn-of-the-year. Journal of Financial Markets. 2003;6(1):73–98.
8. Watkins BD. Institutional Ownership and Return Reversals Following Short-Term Return Consistency. Financial Review. 2006;41(3):435–448.
9. Gutierrez RC, Kelley EK. The Long-Lasting Momentum in Weekly Returns. The Journal of finance. 2008;63(1):415–447.
10. Jegadeesh N, Titman S. Returns to buying winners and selling losers: Implications for stock market efficiency. The Journal of finance. 1993;48(1):65–91.

11. Patel J. Profit from Prices: All You Need for Profit in Stock Trading Is Stock Prices. 1st ed. CreateSpace Independent Publishing Platform; 2007.

12. Figelman I. Stock return momentum and reversal-A comprehensive study. Journal of Portfolio Management. 2007;34(1):51–67.

13. Jegadeesh N, Titman S. Short-horizon return reversals and the bid-ask spread. Journal of Financial Intermediation. 1995;4(2):116–132.

14. Cooper M. Filter rules based on price and volume in individual security overreaction. Review of Financial Studies. 1999;12(4):901–935.

15. Campbell JY, Grossman SJ, Wang J. Trading volume and serial correlation in stock returns. The Quarterly Journal of Economics. 1993;108(4):905–939.

16. Wang J. A model of competitive stock trading volume. Journal of political Economy. 1994; p. 127–168.

17. Da Z, Liu Q, Schaumburg E. Decomposing short-term return reversal. Staff Report, Federal Reserve Bank of New York; 2011.

18. Tang GY, Zhang H. Stock return reversal and continuance anomaly: new evidence from Hong Kong. Applied Economics. 2014;46(12):1335–1349.

19. Hurst HE. Long-term storage of reservoirs: an experimental study. Trans Amer Soc Civil Eng. 1951;116:770–799.

20. Sakalauskas V, Kriksciuniene D. Entropy-Based Indicator for Predicting Stock Price Trend Reversal. In: Business Information Systems Workshops. Springer; 2011. p. 91–98.
